# Nursing work environment and moral sensitivity as factors influencing nurses’ safety behaviors: the mediating role of professional identity

**DOI:** 10.3389/fpubh.2026.1792805

**Published:** 2026-04-01

**Authors:** Shen Liu, Xiaofen Li, Taoyu Lin, Xianqi Gao, Mengting Ai

**Affiliations:** 1 Lishui University School of Medicine, The First Affiliated Hospital of Lishui University, Lishui, China; 2 The First Affiliated Hospital of Lishui University, Lishui People's Hospital, Lishui, China; 3 The People’s Hospital of Suzhou New District, Suzhou, China

**Keywords:** moral sensitivity, nurses, professional identity, safety behavior, work environment of care

## Abstract

**Objective:**

This study is based on the competency-opportunity-motivation-behavior (COM-B) theoretical model to measure the levels of nurses ‘safety behavior, professional identity, moral sensitivity, and nursing work environment, and to explore the relationship between professional identity and nursing work environment and nurses’ safety behavior.

**Methods:**

A cross-sectional survey was conducted among 589 nurses in six public hospitals in Jiangsu and Zhejiang provinces, China. The professional identity scale for nurses, the moral sensitivity questionnaire-revised Chinese version, the Chinese Version of the Nursing Work Environment Scale, and the Chinese version of the nurses’ safety behavior questionnaire were used to measure the level of nurse professional identity, moral sensitivity, nursing work environment, and nurse safety behavior. Person correlation analysis was used to verify the relationships between variables. SEM was used to validate the theoretical hypothesis model.

**Results:**

The total average score of nurses’ safety behaviors was 54.10 ± 7.04, which was above the medium level. In this study, moral sensitivity indirectly affected nurses’ safety behaviors through professional identity (*β* = 0.277, *p* < 0.001, 95%CI = [0.200, 0.371]). Nursing work environment directly affected nurses’ safety behaviors (*β* = 0.239, *p* < 0.001, 95%CI = [0.200, 0.278]) and indirectly affected them through nurse professional identity (*β* = 0.063, *p* < 0.001, 95%CI = [0.015, 0.114]).

**Conclusion:**

This study found that a higher sense of professional identity and a harmonious nursing work environment with adequate support conditions are the foundation for enhancing nurses’ safety behaviors. Hospital administrators and policy makers should provide comfortable working conditions to nurses to increase their professional identity, and should incorporate continuous ethical training into the professional competency development system of nurses to ensure high-quality nursing care and patient safety.

## 1 Introduction

In recent years, the global healthcare system has prioritized patient safety as a key focus (1). The Global Action Plan for Patient Safety 2021–2030 indicates (2) that hospital care in low- and middle-income countries results in 134 million adverse events annually, causing 2.6 million deaths, with nursing errors accounting for over 40% of these incidents. As the primary caregivers during patients’ medical treatment, nurses’ safety behavior directly impact patient safety and the quality of healthcare services delivered by the medical system (3). Research indicates (4) that improving nurses’ safety behavior can reduce the total cost of healthcare by $108 million and save approximately 60,000 inpatient care days. Nurses’ Safety Behavior (NSB) refers to the actions nurses take during daily clinical practice to ensure the safety of patients, themselves, and healthcare institutions. This includes mastering nursing procedures and reporting unsafe behaviors in the workplace (5, 6), such as such as strict adherence to hand hygiene protocols, reporting of adverse nursing events, and performing the three checks and seven verifications for nursing staff. A good nursing work environment can promote the professional identity of nursing staff, help nursing staff to spontaneously improve their safety behavior, and nurses with high level of moral sensitivity are also important. Therefore, it is crucial to clarify the level of nurses’ safety behavior and its influencing factors in order to save medical costs and improve the quality of medical services.

The term “professional identity” originates from the concept of self-identity in psychology (7), which refers to an individual’s sense of belonging and approval toward their profession. Professional identity (8) represents nurses’ self-understanding, encompassing the unique thought patterns and behavioral characteristics of nursing personnel. Relevant studies (9) indicate that nurses with high professional identity are more attentive to patient safety, thereby further promoting safe nursing practices. Ethical sensitivity is a capability that enables nurses to anticipate ethical risks (10), thereby assisting patients in making informed decisions and enhancing patient safety. A study comparing nurses caring for COVID-19 patients demonstrated (11) a significant positive correlation between moral sensitivity and nurses’ safety behaviors. Specifically, nurses with higher moral sensitivity were more likely to perform safety behaviors that benefit patients, thereby improving nursing quality and increasing patient satisfaction. A survey of Iranian intensive care unit (ICU) nurses (12) revealed that nurses’ safety behaviors are not only grounded in professional identity but also require a high level of ethical competence to ensure patient safety. Previous studies have demonstrated (13, 14) that a favorable work environment can provide nurses with support and protection, thereby promoting their positive safety behaviors. However, the influence of work environment on nurses’ safety behaviors may also be moderated by professional identity. Studies have confirmed (15) that a favorable nursing work environment can enhance nurses’ sense of professional value, thereby stimulating their sense of professional achievement.

The Competence-Opportunity-Motivation-Behavior (COM-B) model originated from the theory of behavior change (16), and was proposed by Michie by integrating and drawing on the consensus of relevant behavior theorists (17). The COM-B model comprehensively interprets the underlying mechanisms of behavior, explicitly indicating that behavioral change results from the combined effects of ability, opportunity, and motivation (16). Competence refers to the physiological and psychological capacity of an individual to engage in relevant activities (16). Opportunity is the environment, referring to all external factors that promote behavioral change, including physical and social environments (16). Motivation refers to all process factors that can stimulate and guide brain activity, including clear goals, habitual thinking and working styles, emotional responses, and analytical decision-making, namely automatic and reflective processes (16). Meanwhile, the COM-B model demonstrates that capabilities and opportunities not only directly influence changes in safety behaviors, but also indirectly enhance them by affecting motivation (16). Currently, this theoretical model has been applied in studies to scientifically analyze factors influencing medication safety in patients, improve healthcare providers’ compliance with hand hygiene, and enhance postoperative rehabilitation interventions (18–20), with favorable outcomes achieved.

This study aims to use the competency-opportunity-motivation-behavior (COM-B) model as a conceptual framework, based on the aforementioned literature review, to comprehensively explore the levels of nurses’ safety behaviors and their related factors. The positive cognition of nurses regarding their nursing profession in daily clinical practice constitutes the psychological process that motivates and guides their safe behaviors. This includes both their positive perception of the nursing profession and their reflection on and coping with setbacks encountered in routine work (21). Therefore, professional identity can fully explain the automatic and reflective motivation of nurses’ safety behavior. Currently, extensive research has been conducted on the relationship between professional identity and nurses’ safety behaviors (22, 23), but most studies focus on nursing interns, neglecting the professional cognition of nurses working in high-pressure, complex, and high-workload environments. In the ethical decision-making process, moral sensitivity is defined as nurses ‘understanding of patient vulnerability and their ability to predict the outcomes of patients’ moral decisions, enabling them to make ethical choices for patients (10). Thus, moral sensitivity can serve as a capability to enhance safety behaviors. The nursing work environment encompasses adequate human and material resources in the physical setting, as well as intercollegial collaboration, leadership management styles, and nurses’ involvement in hospital affairs within the social environment (24). Hence, the working environment of nurses can fully represent the motivation that triggers nurses’ safety behavior.

**Figure 1:**
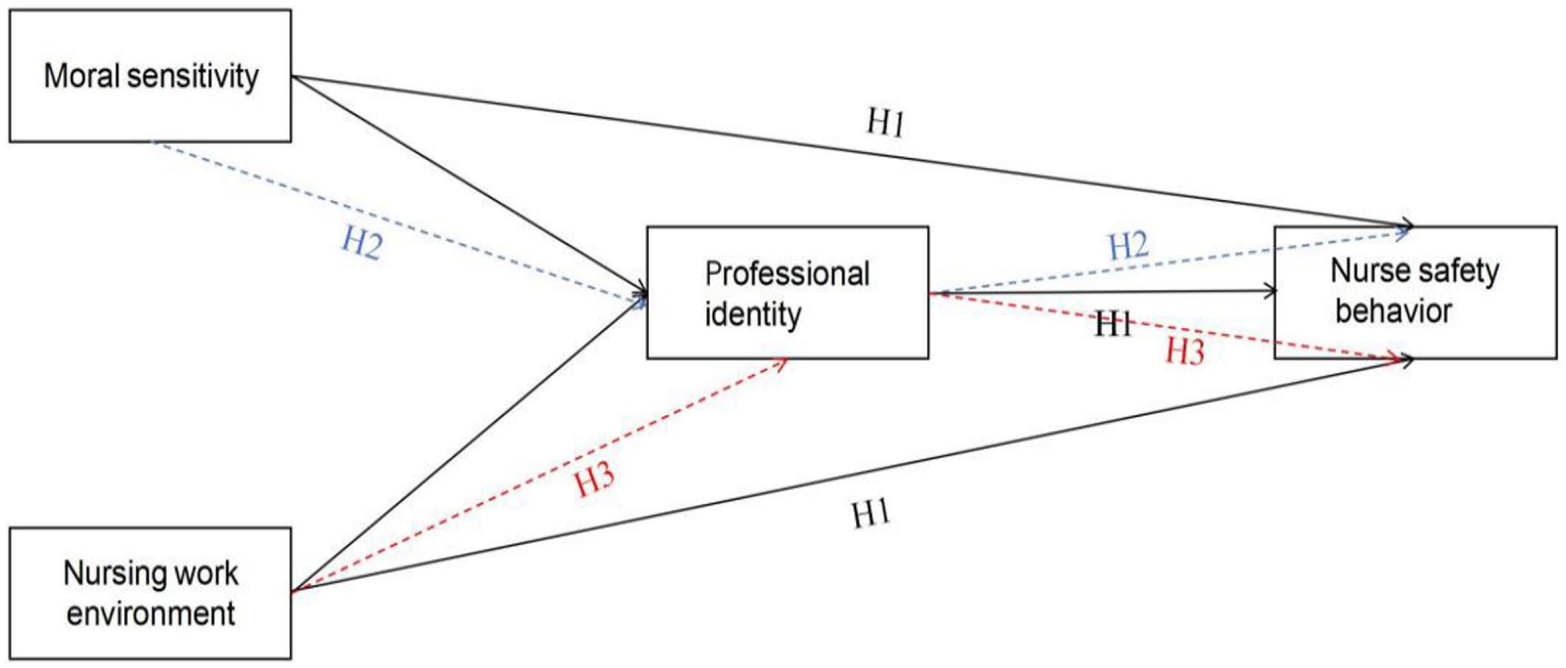
Hypothesis model.

In conclusion, this study employs the COM-B theoretical model to assess nurses ‘safety behavior levels and investigates the mechanisms influencing such behaviors, aiming to provide insights for hospital nursing administrators in developing strategies to enhance nurses’ safety practices. In this study, we proposed the following hypotheses (Figure 1):

(*H1*) Nurses' professional identity, moral sensitivity, and nursing work environment are related to their safety behaviors;

(*H2*) Nurses' professional identity mediates the relationship between moral sensitivity and nurses' safety behaviors;

(*H3*) Nurses' professional identity mediates the relationship between the nursing work environment and nurses' safety behaviors.

## 2 Method

### 2.1 Design

We conducted a descriptive cross-sectional study in accordance with the Strengthening the Reporting of Observational Studies in Epidemiology (STROBE) cross-sectional research report guidelines (25).

### 2.2 Setting and participants

A convenience sampling method was employed, resulting in the recruitment of 590 nurses. Of these, 589 nurses consented to participate and completed the electronic questionnaire via WeChat Questionnaire Star, while one nurse declined participation. The target population comprised 589 nurses employed in public hospitals located in the Jiangsu and Zhejiang provinces of China. Data collection was conducted at six public hospitals within these provinces.

Inclusion criteria were as follows: possession of a nurse practice certificate and registration at the hospital under investigation; active engagement in nursing duties during the study period; employment in the surveyed hospital for more than six months; and voluntary participation. Exclusion criteria included nurses on leave or off duty during data collection and nurses from other hospitals temporarily present at the survey site for study or training.

Based on the sample size estimation principle for multi-factor analysis (26), the required sample size should be 10 to 20 times the number of observed variables. Allowing for a 20 percent rate of invalid responses, the estimated sample size for this study ranged from 372 to 744 participants.

### 2.3 Instruments/measures

#### 2.3.1 Demographic characteristics

The demographic characteristics comprised 13 variables: age, gender, education level, employment status, nursing experience, work-related income level, family situation, marital status, number of night shifts, job satisfaction, and frequency of monthly safety training sessions for nurses.

#### 2.3.2 Nurses’ safety behaviors

We used the Chinese version of the Nurses’ Safety Behaviors Questionnaire (NSBQ) to measure nurses’ safe behavior levels. The questionnaire was originally developed by Shih (27) and subsequently translated by Rong (28). This questionnaire is a single dimension, including 12 items, each of which is scored on a 5-point Likert scale. The higher the total score, the better the safety behavior performance of nurses. In this study, Cronbach’s *α* was 0.961.

#### 2.3.3 Nurses’ professional identity

This study used the professional identity scale (NPI) for nurses to investigate the status quo of nurses’ vocational identity. Developed by Liu Ling (29) based on China’s nursing practice realities, this 30-item scale evaluates five dimensions: professional identity evaluation, occupational social support, social skills in the workplace, coping with career setbacks, and professional self-reflection. Using a 5-point Likert scale (ranging from “strongly disagree” to “strongly agree”), higher scores indicate stronger professional identity. The scale had good reliability and validity, with a Cronbach’s *α* value of 0.983 in this study.

#### 2.3.4 Moral sensitivity

The Moral Sensitivity Questionnaire-Revised Version into Chinese (MSQ-R-CV) was developed by Lützen (30). Huang (31) revised the original scale and used it to evaluate the moral sensitivity of nurses in China. The scale has two dimensions: moral responsibility and power, and sense of moral burden, with nine items. The Likert 6-point scoring method (1 = I do not agree entirely, 2 = do not agree completely, 3 = uncertain, 4 = basically agree, 5 = relatively agree, 6 = I agree entirely) is adopted. The higher the total score, the higher the moral sensitivity of nurses. In this study, the Cronbach’s *α* of the scale was 0.871.

#### 2.3.5 Nursing work environment

The nursing work environment was reflected by the practice environment scale of the nursing work (PES). The scale was originally developed by Lake (32) and it was translated and revised by Wang (33). The scale consists of five dimensions: nurses’ engagement in hospital operations, the foundation of high-quality nursing services, leadership capabilities and approaches of nursing managers, adequacy of human and material resources, and medical-nursing collaboration, with 31 items in total. The scale comprises five dimensions: nurses’ engagement in hospital operations, the foundation of high-quality nursing services, leadership capabilities and approaches of nursing managers, adequate human and material resources, and medical-nursing collaboration, with 31 items in total. Using a Likert 4-point scale (1–4 points for “strongly disagree” to “strongly agree”), higher scores indicate better working environments for nurses. In this study, the Cronbach’s *α* of the scale was 0.982.

### 2.4 Data collection

Data were distributed and collected using the WeChat online questionnaire platform between April and August 2025, with support from nursing managers. Head nurses disseminated the questionnaire to departmental work groups using a QR code. The study’s objectives, voluntary participation, and confidentiality measures were communicated during meetings and included in the QR code link. Before distribution, head nurses received training on survey procedures. The informed consent form described the study’s objectives and confirmed that participation was anonymous and voluntary. Only nurses who selected the informed consent option were able to proceed with the questionnaire. No personal identifying information was collected to protect the respondent’s privacy. All data is stored on a secure, password-protected server. Completion of the questionnaire requires approximately 15 to 20 min. Invalid responses will be excluded based on the following criteria: completion in less than 5 min, identical answers to all questions, or missing items. Data processing is conducted in accordance with the EU General Data Protection Regulation (34) (GDPR).

### 2.5 Statistical analysis

IBM SPSS 26.0 and AMOS 28.0 software were utilized for statistical analysis. The Shapiro–Wilk test assessed the normality of continuous data. Descriptive statistics, including percentages, frequencies, means, and standard deviations, summarized the data. One-way ANOVA was conducted to test intergroup differences, followed by *post hoc* comparison analysis. Pearson correlation analysis examined the relationships between nurses’ safety behaviors and professional identity, moral sensitivity, and the nursing work environment, with statistical significance set at 0.05. AMOS 28.0 was employed to verify the mediating effects of professional identity, moral sensitivity, and the nursing work environment on nurses’ safety behaviors.

The two-stage method of structural equation modeling (35) was applied to assess model fit and verify the hypothesized model. Model fit was evaluated using the following statistical indicators (36, 37): the ratio of chi-square value to degrees of freedom (*χ*^2^/df), comparative fit index (CFI), Tucker-Lewis index (TLI), normative fit index (NFI), and root mean square error of approximation (RMSEA). The mediation effect of vocational identity was tested using the self-replication method. Statistical significance was defined as *p* < 0.05 (two-tailed).

### 2.6 Ethical consideration

The study received approval from the Ethics Committee of the Medical School of *** (ethical review number: 2025R013). Before data collection, the researchers informed participants about the survey’s purpose and methodology, the principles of voluntary and anonymous participation, and participants’ rights. Furthermore, no names or personal information will be disclosed to ensure participant privacy.

## 3 Results

### 3.1 Demographic characteristics

A total of 590 questionnaires were collected, with one explicit refusal to participate, resulting in an effective response rate of 99.8%. Of the respondents, 515 (87.4%) worked in tertiary hospitals. Among the 589 nurses included in the analysis, 327 (55.5%) were aged 26 to 35 years, and 546 (92.7%) were female. Most respondents (499, 84.7%) held a bachelor’s degree, and the majority were married (355, 60.3%). Notably, 297 nurses (50.4%) had more than 10 years of work experience. Additionally, 306 nurses (52.0%) held intermediate or higher professional titles, and 498 (84.6%) had an N2 or higher ability level. Table 1 presents the safety behavior scores and complete demographic characteristics of the nurses.

**Table 1:** Participants’ characteristics and the median of the nurse safety behavior (*n* = 589).

Variable	*n* (%)	Nurse safety behavior	*t*	*p*
		Mean ± SD		
Region			1.60	0.20
Xuzhou	78 (13.2)	55.42 ± 6.93		
Suzhou	44 (7.5)	54.09 ± 6.62		
Lishui	467 (79.3)	53.88 ± 7.09		
Hospital level			1.44	0.42
tertiary hospitals	515 (87.4)	54.30 ± 6.69		
Secondary hospital	74 (12.6)	53.14 ± 9.33		
Gender			28.60	*p* < 0.01
Male	43 (7.3)	48.70 ± 9.48		
Female	546 (92.7)	54.53 ± 6.64		
Age (years)			8.37	*p* < 0.01
≤25	95 (16.1)	51.90 ± 7.26		
26–30	171 (29.0)	52.88 ± 7.38		
31–35	156 (26.5)	54.63 ± 6.15		
36–40	93 (15.8)	55.29 ± 8.19		
>40	74 (12.6)	57.16 ± 4.29		
Marital status			12.78	*p* < 0.01
Married	355 (60.3)	55.23 ± 6.66		
Single	223 (37.9)	52.26 ± 7.28		
Other marital status	11 (1.8)	55.09 ± 7.17		
Education level			3.07	0.027
Diploma	81 (13.8)	52.95 ± 8.07		
Bachelor	499 (84.7)	54.39 ± 6.82		
Master and above	9 (1.5)	48.33 ± 7.35		
Work experience in nursing			19.02	*p* < 0.01
Less than 5 years	143 (24.3)	51.42 ± 7.80		
5–10 years	149 (25.3)	53.60 ± 6.50		
More than 10 years	297 (50.4)	55.65 ± 6.50		
Nursing title			7.79	*p* < 0.01
Nurse	89 (15.1)	51.19 ± 8.45		
Senior nurse	194 (32.9)	53.35 ± 6.57		
Charge nurse	239 (40.6)	55.20 ± 6.88		
Associate chief nurse and above	67 (11.4)	56.22 ± 5.24		
Employment status			6.65	*p* < 0.01
Formal establishment	296 (50.3)	54.63 ± 6.87		
Contractual employment	218 (37.0)	53.50 ± 6.95		
Personnel agency	62 (10.5)	55.27 ± 5.52		
Others	13 (2.2)	46.70 ± 12.74		
Nursing competency level			8.93	*p* < 0.01
*N*0	46 (7.8)	50.59 ± 8.43		
*N*1	45 (7.6)	51.29 ± 8.62		
*N*2	275 (46.7)	53.73 ± 6.40		
*N*3	166 (28.2)	55.63 ± 7.16		
*N*4	57 (9.7)	56.51 ± 4.90		
Nurses’ income level(yuan)			1.98	0.12
<4,000	51 (8.7)	52.25 ± 9.04		
4,001–6,000	266 (45.1)	53.85 ± 7.11		
6,001–8,000	196 (33.3)	54.78 ± 6.08		
>8,000	76 (12.9)	54.50 ± 7.49		
Nurses’ job satisfaction			28.82	*p* < 0.01
Very dissatisfied	15 (2.5)	48.73 ± 14.39		
Somewhat dissatisfied	84 (14.3)	50.01 ± 8.77		
Somewhat satisfied	374 (63.5)	53.98 ± 6.22		
Very satisfied	116 (19.7)	58.16 ± 3.84		
Frequency of safety knowledge training (times/month)			11.97	*p* < 0.01
0	38 (6.5)	49.13 ± 9.55		
1–2	397 (67.4)	54.13 ± 6.70		
>2	154 (26.1)	55.26 ± 6.72		

### 3.2 Correlations between variables

The Pearson correlation test was used to test the correlation between variables. The analysis results showed that nurses’ safety behavior was significantly positively correlated with moral sensitivity (*r* = 0.322, *p* < 0.001), professional identity (*r* = 0.569, *p* < 0.001), and nursing working environment (*r* = 0.655, *p* < 0.001) (Table 2).

**Table 2:** Correlation between moral sensitivity, professional identity, nursing work environment, and patient safety behavior (*n* = 589).

Variable	Nurse safety behavior	Moral sensitivity	Professional identity	Nursing work environment
Nurse safety behavior	1			
Moral sensitivity	0.322^**^	1		
Professional identity	0.560^**^	0.548^**^	1	
Nursing work environment	0.655^**^	0.403^**^	0.706^**^	1

^*^Significant at *p* < 0.05, ^**^Significant at *p* < 0.01.

### 3.3 Structural equation model

**Figure 2:**
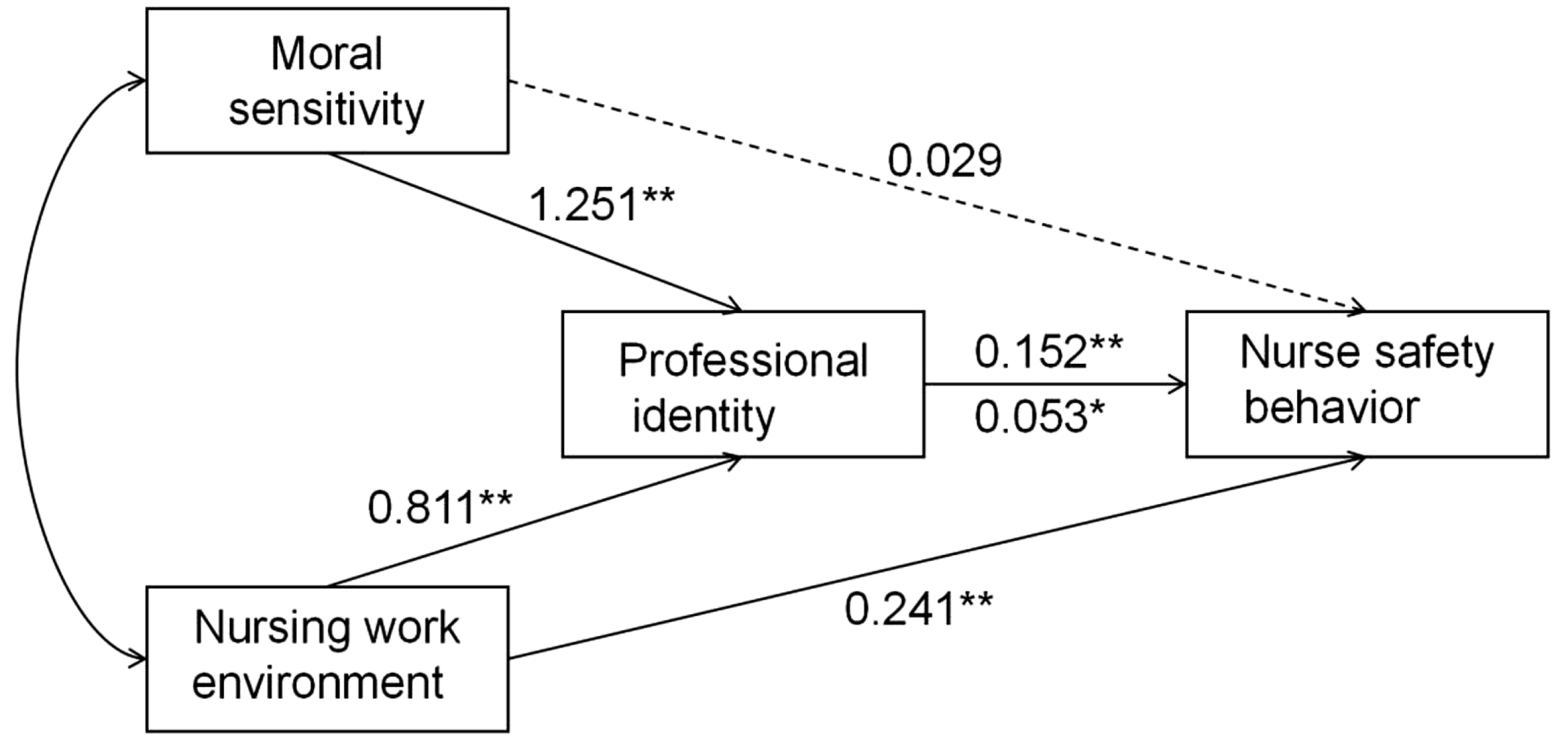
Pathway relations and effect values for the hypothetical model. The solid lines represent *p* < 0.05, and the dotted line represents *p* > 0.05. ^*^Significant at *p <* 0.05, ^**^Significant at *p <* 0.01.

We used a structural equation model to examine the relationships among professional identity, moral sensitivity, nursing work environment, and nurses’ safe behavior. Variables with significant correlations were analyzed using SPSS Process 4.1 to test for mediating effects. Key demographic characteristics, such as gender, age, education, professional title, energy level, employment mode, marital status, years of experience, night shift frequency, job satisfaction, and safety training frequency, were included as covariates. The model results, shown in Figure 2, differ from the initial assumptions. Model fit indices were *χ*^2^/df = 6.370, CFI = 0.905, TLI = 0.895, NFI = 0.889, and RMSEA = 0.096. Path terms without statistical significance were set to zero for model modification, yielding a modified model with better fit: *χ*^2^/df = 4.540, CFI = 0.954, TLI = 0.947, NFI = 0.941, and RMSEA = 0.078.

We conducted a bootstrap mediation analysis with 5,000 iterations to assess whether vocational identity mediates the effects of moral sensitivity and the nursing work environment on nurse safety behavior. Results indicated that professional identity significantly mediated the relationship between ethical sensitivity and nurse safety behavior (*β* = 0.190, *p* < 0.001, 95% CI = [0.128, 0.261]). Vocational identity also mediated the relationship between the nursing work environment and patient safety behaviors (*β* = 0.043, *p* < 0.001, 95% CI = [0.004, 0.089]) (Table 3).

**Table 3:** The mediating effect of professional identity between risk perception, moral sensitivity, nursing work environment, and nurse safety behavior, and 95% confidence intervals.

Paths		Effect	*p*	Boot SE	95% CI [Lower, Upper]
Direct effect	Moral sensitivity → nurse safety behavior	0.029	0.433	0.037	[−0.044, 0.102]
Indirect effect	Moral sensitivity → professional identity → nurse safety behavior	0.190	<0.001	0.034	[0.128, 0.261]
Total effect		0.219	<0.001	0.034	[0.151, 0.286]
Direct effect	Work environment → nurse safety behavior	0.241	<0.001	0.020	[0.202, 0.279]
Indirect effect	Work environment → professional identity → nurse safety behavior	0.043	<0.001	0.022	[0.004, 0.089]
Total effect		0.283	<0.001	0.016	[0.252, 0.315]

## 4 Discussion

Drawing on the capability opportunity motivation-behavior (COM-B) model, this study validated the conceptual relationships among moral sensitivity (psychological ability), nursing work environment (opportunity), and nurses’ safety behavior (behavior), with career identity representing motivation. The structural equation modeling results largely support the hypothesized model: nurses with high moral sensitivity demonstrate a stronger sense of professional identity, which in turn increases their safety behavior. Additionally, managerial provision of adequate work resources and support can foster professional identification among nurses, thereby enhancing their safety behavior.

The findings indicated that the average score for nurses’ safety behaviors exceeds the midpoint, aligning with previous research that reports a high level of safety behavior among nurses (38). This outcome may be attributed to the extensive clinical experience, long working hours, and strong competencies of the surveyed nurses (39, 40). However, other studies suggested that prolonged working hours, high job demands, and the inherent pressures of the nursing profession can lead to job burnout, which negatively impacts work quality and patient safety (41). Consequently, hospital managers should not only monitor nurses’ safety behavior but also ensure a supportive and secure work environment for frontline nurses facing heavy workloads and high stress, in order to mitigate burnout. Implementing effective human resource strategies, such as optimizing night shift rotations and minimizing unnecessary meetings, can reduce nurse turnover, alleviate stress and fatigue, and ultimately promote safer behaviors.

The survey results supported the first hypothesis (H1) of the COM-B theoretical model, indicating that nurses’ safety behavior is associated with professional identity, moral sensitivity, and the nursing work environment. Notably, the correlation between moral sensitivity and safety behaviors was substantially lower than the correlations involving professional identity and work environment. This may be due to the predominance of experienced nurses in the sample, who may experience heightened job burnout from sustained high pressure, risk, and workload, potentially diminishing their moral sensitivity (42). This finding contrasts with previous studies that identified a significant positive correlation between moral sensitivity and nurses’ safety behaviors (11). One possible explanation is that during the COVID-19 pandemic, nurses may have unconsciously heightened their moral sensitivity, leading to more meticulous patient care (11). The observed relationship between the nursing work environment and safety behaviors is consistent with prior research (14), which demonstrates that nurses in well-resourced, supportive environments provide more efficient care. Such efficiency enhances overall nursing quality and patient outcomes, thereby fostering higher levels of safety behavior (13). Furthermore, strengthening nurses’ professional identity can improve job satisfaction, which in turn enhances nursing quality and safety behaviors (43).

The findings supported the COM-B theoretical model, indicating that professional identity mediates the relationships between both moral sensitivity and safety behavior (H2) and the nursing work environment and safety behavior (H3). These results suggest that nurses with higher moral sensitivity may spontaneously reflect on their professional roles and responsibilities, thereby enhancing their professional identity and job engagement, which in turn improves their safety behavior levels. A well-designed nursing environment could enhance nurses’ sense of professional identity, thereby fostering a sense of professional achievement. Nurses with a strong sense of professional identity are more likely to prioritize patient safety, which further promotes their safety behaviors. Our findings are consistent with previous studies (9, 44). Sahar (44) found that improving moral competence enhances nurses’ understanding of their profession and supports nursing quality. The COM-B model also highlights the importance of career identity and values in career development (45). The development of moral competence would enhance nurses’ awareness of professional identity and deepen their sense of professional identity. Professional identity not only enables nurses to accurately identify ethical issues but also plays a crucial role in recognizing workplace risk factors to ensure patient safety (45). However, in this study, there was no direct relationship between moral sensitivity and safety behavior. This result is biased from the previous results, which may be because the institutional norms of our country’s hospitals regulate the ethical principles of nursing staff, thus weakening the relationship between moral sensitivity and safety behavior. Furthermore, the nursing safety behavior measurement tool used in this study only represents the basic safety behaviors in daily nursing work, and does not include dimensions such as ethical decision-making.

Nursing managers should regularly assess nurses’ physical competencies and support the development of their psychological capabilities. Triage nurses serve as the initial point of contact for patients and are responsible for patient care during triage, verifying patient identity, assessing severity and safety, and managing patient emotions (46). Establishing a standardized triage process, fostering a supportive nursing environment, and utilizing effective communication techniques can help patients feel secure and encourage them to accurately report their health status, thereby enhancing both patient experience and nurses’ adherence to safety protocols (47). Furthermore, a positive work environment, characterized by strong collegial cooperation, adequate staffing and resources, leadership support, and opportunities for participation in hospital affairs, can significantly increase nurses’ motivation to engage in safe practices (32). In recent years, young nurses have faced unstable working environments while receiving relatively standardized nursing ethics education (48). This has led them to spontaneously maintain strong critical thinking by adhering to ethical norms, enabling them to rapidly identify patient needs. Accordingly, nursing administrators should incorporate the continuous professional competency development of young nurses into their core management agenda. By establishing systematic training strategies, they can maintain and enhance the high-level risk perception and judgment capabilities of the nursing workforce, thereby providing essential support for improving the quality of clinical nursing safety and ethical decision-making. Five European countries have established the European Mentorship System, which provides a supportive nursing environment for novice nurses and nursing interns by setting mentors as role models, thereby facilitating their rapid professional development (49). Therefore, nursing administrators can establish a mentorship collaboration system to guide novice nurses in reflecting on the shortcomings of their teaching methods, thereby continuously promoting their self-discovery and leadership skill development, with the aim of advancing their mentorship role growth. Furthermore, hospitals should provide 24-h ethical counseling services for frontline nursing staff to enhance the cultivation of nurses’ moral sensitivity (50).

Our results were inconsistent with the hypothesized model, as we found no significant direct link between moral sensitivity and nurse safety behavior. This echoes the work of Huang (45), who believed that the nurse safety behavior scale used in our study focused only on basic daily safety actions and overlooked more complex ethical decisions, which may have influenced the results. Looking ahead, using a more comprehensive, multi-dimensional safety behavior scale and increasing the sample size could help clarify the relationship between moral sensitivity and safety behavior. It is also possible that moral sensitivity reflects only the psychological aspect of ability in the COM-B theory framework. Future research should explore how both psychological and physical competence together shape nurses’ safety behavior.

### 4.1 Limitations

Our study has several limitations. First, the sample was limited to experienced nurses from the eastern coastal regions of China, which may not represent the diversity of safety behaviors among nurses in other regions. This limitation may affect the generalizability of our findings. Second, although this study was guided by theoretical principles, the cross-sectional design cannot establish causal relationships regarding nurses’ safety behaviors. Furthermore, the data collected in this study were obtained through self-administered questionnaires designed solely to assess nurses’ basic safety behaviors, which may introduce subjectivity and measurement bias, potentially compromising the reliability and accuracy of the findings. Future studies could consider incorporating objective data to reduce bias in results, and address the limitations of this study through longitudinal tracking or randomized controlled trial designs.

## 5 Conclusion

Nursing managers should prioritize enhancing nurses’ moral sensitivity and offer flexible opportunities for ongoing ethics education, integrating these into existing training programs. This approach can strengthen nurses’ sense of responsibility, professional belonging, and commitment to safe practices (51). Additionally, fostering an autonomous, open, inclusive, and supportive environment can further reinforce professional identity and safety behaviors. Future research can apply the COM-B theoretical model to identify factors influencing nurses’ safety behaviors and use the behavioral change wheel to develop systems that align with clinical practice, ultimately improving patient safety and nursing quality. This study offers valuable insights for advancing research on nurses’ safety behaviors.

## Data Availability

The datasets presented in this article are not readily available because the data involves personal privacy and will not be provided. Requests to access the datasets should be directed to liushen0731@163.com.
